# Efficacy and safety of insulin icodec compared with insulin efsitora in patients with type 2 diabetes: a systematic review and network meta-analysis

**DOI:** 10.3389/fendo.2026.1902260

**Published:** 2026-08-14

**Authors:** Jingxin Li, Junxia Wen, Hao Zheng, Cuilu Quan, Jiajie Bi, Yajing Yang, Jing Zhang, Yanhong Shen

**Affiliations:** Department of Pharmacy, Hebei Chest Hospital, Shijiazhuang, China

**Keywords:** insulin efsitora, insulin icodec, network meta-analysis, once-weekly insulin, systematic review, type 2 diabetes

## Abstract

**Objective:**

We conducted this network meta-analysis to compare the efficacy and safety of insulin icodec vs insulin efsitora for patients with type 2 diabetes (T2D).

**Methods:**

We conducted a comprehensive systematic search across PubMed, the Cochrane Library, and Embase up to November 11, 2025. Eligible trials compared insulin icodec and insulin efsitora against each other, placebo or other glucose-lowering drugs. Primary outcome was change in HbA1c. Secondary outcomes were change in body weight, percentage of patients achieving HbA1c < 7%, change in fasting plasma glucose (FPG), and safety outcomes including level 1 hypoglycemia, level 2 or level 3 hypoglycemia, adverse events (AEs), and serious adverse events (SAEs). We used version 2 of the Cochrane risk-of-bias tool to assess the risk of bias. A frequentist network meta-analysis was used to calculate the risk ratios or mean difference with corresponding 95% confidence intervals.

**Results:**

Thirteen RCTs involving 7,402 participants were included. Insulin icodec resulted in a lower HbA1c level compared to insulin efsitora, but was associated with a higher incidence of level 1 hypoglycemia. The two treatments showed no significant differences in other outcomes, including change in body weight, percentage of patients achieving HbA1c <7%, change in FPG, incidence of level 2 or 3 hypoglycemia, AEs, and SAEs. Compared to daily insulins, insulin icodec led to lower HbA1c levels. Weight gain was lower with insulin glargine than with insulin icodec. A higher percentage of patients in the icodec group achieved HbA1c <7% compared to those receiving daily insulins. Regarding safety outcomes, insulin degludec had a lower incidence of both level 1 and level 2 or 3 hypoglycemia than the other three insulins. Insulin icodec showed a higher incidence of level 1 hypoglycemia than insulin glargine. Additionally, the incidence of AEs was higher with insulin icodec than with insulin degludec.

**Conclusions:**

In patients with T2D, once-weekly insulin icodec may provide slightly better glycaemic control than daily insulin. Insulin icodec was more effective than insulin efsitora in reducing HbA1c levels; however, this benefit was offset by a significantly higher risk of level 1 hypoglycemia. The two insulins were comparable in terms of body weight change, glycemic target achievement, FPG reduction, and safety profiles, including level 2 or 3 hypoglycemia, AEs, and SAEs. Direct head to head RCTs are warranted to confirm these findings.

**Systematic review registration:**

https://www.crd.york.ac.uk/PROSPERO/, identifier CRD420251177019.

## 1 Introduction

Type 2 diabetes (T2D) is a chronic metabolic disorder characterized by progressive β-cell dysfunction and insulin resistance, affecting an estimated 537 million adults worldwide in 2021, with the number projected to rise to 783 million by 2045 (1). Achieving and maintaining glycaemic control is central to reducing the risk of long-term microvascular and macrovascular complications. However, the progressive nature of T2D means that many patients eventually require exogenous insulin therapy to meet glycaemic targets. Despite the well-established efficacy of basal insulin, adherence to daily injection regimens remains a substantial challenge (2, 3). The frequency of injections—365 or more per year—can lead to injection fatigue, reduced quality of life, and ultimately suboptimal glycaemic outcomes (4, 5).

In recent years, the development of once-weekly basal insulin formulations has emerged as a promising strategy to reduce injection burden and potentially improve treatment adherence (6, 7). By reducing the annual number of basal insulin injections from 365 to 52, these novel agents offer the potential to simplify diabetes management without decreasing glycaemic efficacy. Two once-weekly basal insulin candidates have advanced through comprehensive phase 3 clinical development programmes: insulin icodec (Novo Nordisk) and insulin efsitora alfa (Eli Lilly).

Insulin icodec is a basal insulin analogue engineered for extended half-life, enabling once-weekly subcutaneous administration (6). Its phase 3 development programme, ONWARDS, comprised six randomised controlled trials (RCTs) evaluating icodec across diverse populations of patients with T2D, ranging from insulin-naïve individuals to those on basal-bolus regimens (8–13). Insulin efsitora alfa is a novel once-weekly basal insulin that combines a single-chain insulin variant with a human IgG2 Fc domain, resulting in a prolonged half-life and a low peak-to-trough ratio that aims to provide stable glycaemic control throughout the weekly dosing interval (14). The phase 3 QWINT (Once Weekly Insulin Therapy) programme included five RCTs evaluating efsitora across the spectrum of T2D management-from insulin-naïve patients (QWINT-1 and QWINT-2) to those previously treated with basal insulin (QWINT-3 and QWINT-4), as well as a trial in type 1 diabetes (QWINT-5) (15–19). Meta-analysis results have also shown that two weekly insulin preparations are similar to daily formulations in both safety and efficacy (20, 21). Taken together, the ONWARDS, and QWINT programme has established weekly insulin as an effective alternative to daily basal insulin, with a favourable hypoglycaemia profile in certain populations.

Despite the individual efficacy and safety evidence for icodec and efsitora, no head-to-head RCT has directly compared these two once-weekly insulin formulations. In the absence of direct comparative evidence, clinicians and healthcare decision-makers must rely on indirect comparisons to guide treatment choices between these two novel agents. Given the growing clinical interest in once-weekly insulins and the lack of direct comparative data, a systematic network meta-analysis that integrates both direct and indirect evidence from all available RCTs comparing icodec and efsitora with common comparators (daily basal insulins) is warranted. Accordingly, we conducted a network meta-analysis to compare the efficacy and safety outcomes of once-weekly insulin icodec versus once-weekly insulin efsitora in adults with T2D. The findings of this network meta-analysis aim to inform clinical decision-making by providing the most comprehensive and up-to-date comparative evidence between these two once-weekly insulin formulations.

## 2 Materials and methods

This network meta-analysis was conducted in accordance with the Preferred Reporting Items for Systematic Reviews and Meta-Analyses (PRISMA) extension statement for network meta-analyses (22). The protocol was registered in the PROSPERO database (registration ID: CRD420251177019) and followed the standard methodological guidance for conducting systematic reviews and network meta-analyses of RCTs.

### 2.1 Search strategy and selection criteria

A comprehensive systematic literature search was performed in the following electronic databases from inception to November 11, 2025: PubMed, the Cochrane Library, and Embase. The search strategy was developed using a combination of Medical Subject Headings (MeSH) and free-text terms including: “insulin icodec”, “NN1436”, “efsitora”, “LY3209590”, “basal insulin Fc”, “BIF”, “type 2 diabetes”, “T2D”, “randomized controlled trial”, and “RCT”. No language or date restrictions were applied. Additionally, reference lists of included studies and relevant review articles were manually screened to identify any additional eligible trials. Conference proceedings from major diabetes meetings were also searched for unpublished or ongoing trials.

Studies were included if they met the following criteria: Design: RCTs; Population: Patients with T2D; Intervention: Insulin icodec or insulin efsitora; Comparator: Placebo, once daily basal insulin or other antidiabetic drugs; Outcomes: Efficacy outcomes: change in HbA1c, change in body weight, percentage of patients achieving HbA1c < 7%, change in fasting plasma glucose (FPG). Safety outcomes: Level 1 hypoglycemia, level 2 or level 3 hypoglycemia, adverse events (AEs), and serious adverse events (SAEs). We excluded case reports, cohort studies, conference abstracts, letters, comments, duplicate studies, and RCTs with insufficient or unusable data. We also excluded the RCTs which involved minors, gestational diabetes or other special diabetic groups. Two independent reviewers conducted the study selection. Titles and abstracts were screened to exclude irrelevant studies. Potentially eligible articles underwent full-text assessment based on predefined inclusion and exclusion criteria. Any discrepancies between reviewers were resolved through discussion with a third researcher to reach consensus.

### 2.2 Data extraction and risk of bias assessment

A standardized data extraction form was developed and piloted prior to use. Two reviewers independently extracted the following information from each included study: Study and participant characteristics: Trial name, publication year, study arms, sample size, mean age, sex distribution, body weight, baseline body mass index (BMI), mean baseline HbA1c, duration of diabetes, treatment duration.

Outcome data: For continuous outcomes (change in HbA1c, change in body weight, change in FPG), mean change from baseline to end of study with standard deviation were extracted. If standard deviation was not reported directly, it was calculated from confidence intervals (CIs), standard errors, p-values, or interquartile ranges where possible. For dichotomous outcomes (percentage of patients achieving HbA1c < 7%, level 1 hypoglycemia, level 2 or level 3 hypoglycemia, AEs, and SAEs), the number of participants experiencing the event and the total number of participants in each treatment arm were extracted.

Discrepancies in data extraction were resolved by consensus or by a third reviewer. When data were missing or incomplete, attempts were made to contact corresponding authors or to retrieve data from ClinicalTrials.gov or supplementary appendices.

Two independent reviewers assessed the risk of bias for each included study using the version 2 of the Cochrane risk-of-bias tool (RoB 2), as recommended by the Cochrane Handbook for Systematic Reviews of Interventions. The following domains were evaluated: Randomization process; deviations from intended interventions; missing outcome data; measurement of the outcome; selection of the reported result. Each domain was rated as “low risk of bias”, “some concerns”, or “high risk of bias”. An overall risk of bias judgment was then assigned for each outcome of interest. A study was considered to have low risk of bias if all domains were rated as low risk; some concerns if at least one domain had some concerns but no high-risk domains; and high risk of bias if any domain was rated as high risk or if multiple domains had some concerns that substantially increased the risk of bias. Disagreements between reviewers were resolved by discussion or by consulting a third reviewer. The risk of bias assessment results were presented as summary figures using the RoB 2 visualization tool. Finally, we assigned recommendation grades to the studied outcomes using the Grading of Recommendations Assessment, Development, and Evaluation (GRADE) framework. Two authors independently evaluated evidence quality, with disagreements settled through discussion.

### 2.3 Statistical analysis

We performed a frequentist network meta-analysis using Stata 16.0 (StataCorp LP, College Station, TX, USA). For continuous outcomes, we calculated mean differences (MD) with 95% CIs to account for variations across studies. For dichotomous variables, treatment effects were expressed as risk ratio (RR) with 95% CIs. If the 95% CI of the RR did not contain 1, the difference was considered statistically significant.

Network diagrams were generated to visualize the relationships between interventions, where the size of each node represents the sample size of the corresponding intervention, and the thickness of the edges indicates the number of studies directly comparing the two interventions. A consistency model was adopted if no significant global inconsistency was detected based on the design-by-treatment interaction model (*p*≥0.05); otherwise, an inconsistency model was considered. When a closed loop existed, the Z-test was employed for consistency evaluation, with *p*>0.05 suggesting significant consistency. To further quantify local inconsistency, a node-splitting analysis was performed. Given presumed clinical heterogeneity across studies, all primary NMA analyses were conducted using a random-effects model.

The surface under the cumulative ranking curve (SUCRA) was used to assess and rank the effectiveness of each intervention; higher SUCRA values indicated better efficacy. SUCRA values represent relative ranking probabilities rather than formal statistical tests of superiority; therefore, interpretation of small differences between interventions must account for overlapping CIs and the overall uncertainty of effect estimates. A hierarchical ranking diagram of efficacy and safety was also produced to visually present the results. Publication bias and small-study effects were evaluated using comparison-adjusted funnel plots. All statistical analyses were performed using Stata 16.0.

## 3 Results

### 3.1 Study selection and characteristics

**Figure 1:**
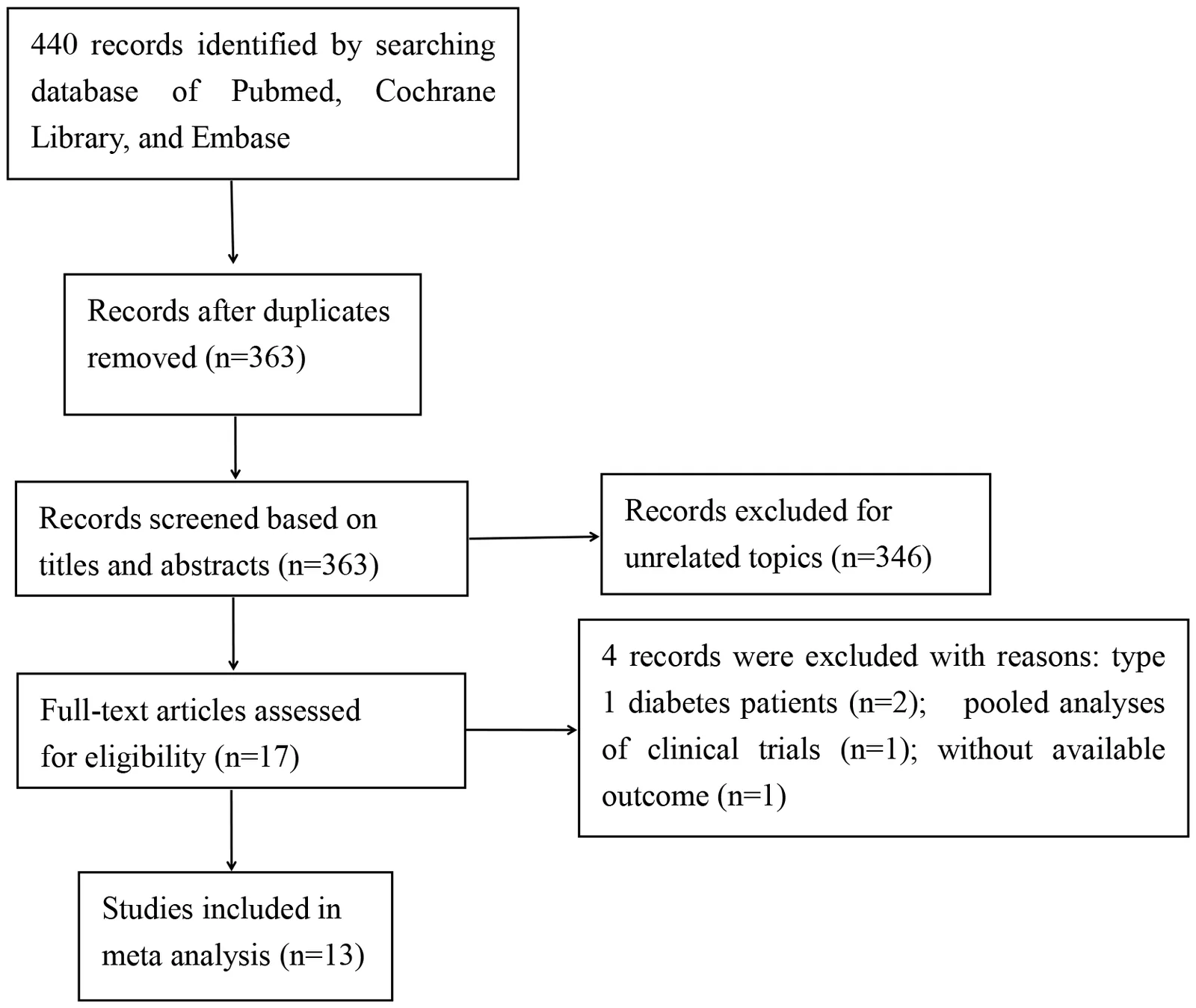
The selection process of included studies.

A total of thirteen RCTs (8–11, 15–18, 23–27) comprising 7,402 participants with T2D were included in this systematic review and network meta-analysis. The study selection process is presented in **Figure 1**. As shown in **Table 1**, the included studies enrolled a relatively heterogeneous population across 13 RCTs, with mean baseline HbA1c ranging from 7.7% (QWINT-3) to 8.55% (ONWARDS 3), mean age from 56.2 to 62.6 years, mean body weight from 81.5 kg to 91.3 kg, and mean diabetes duration from 8.8 to 18.0 years. Notably, ONWARDS 3 and QWINT-3 enrolled patients with the longest diabetes duration (over 14 years) and the lowest baseline HbA1c (around 7.7%), while ONWARDS 1 and QWINT-2 included patients with higher baseline HbA1c (>8.2%) and relatively shorter diabetes duration. Despite these variations, baseline characteristics were generally well balanced between treatment arms within each individual trial, and background glucose-lowering therapies were comparable across arms.

**Table 1:** Characteristics of the included studies and patients.

Trial name	Country	Study arms	Patients	Age (years)	Male, n (%)	Bodyweight (kg)	BMI (kg/m^2^)	HbA1c (%)	Duration of diabetes (years)	Treatment duration (weeks)
QWINT-2 2024	Brazil, Canada, China, et al	Efsitora	466	57.6 ± 10.6	281 (60.3)	86.83 ± 20.53	30.44 ± 5.85	8.21 ± 0.96	11.78 ± 7.54	52
		Degludec	462	57.3 ± 11.0	265 (57.4)	86.12 ± 18.93	30.72 ± 5.90	8.23 ± 0.96	11.42 ± 6.97	
Bue-Valleskey2023	Argentina, Germany, Poland, et al	Efsitora	143	57.3 ± 9.7	76(53.1)	91.0 ± 20.8	32.3 ± 5.4	8.1 ± 0.8	10.4 ± 6.8	26
		Degludec	135	59.4 ± 9.1	76(56.3)	90.6 ± 19.6	31.6 ± 5.5	8.0 ± 0.8	9.7 ± 6.0	
Frias2023	USA, Puerto Rico, and Mexico	Efsitora	267	59.9 ± 10.6	130(49)	89.4 ± 19.2	32.5 ± 5.9	8.1 ± 0.9	14.6 ± 8.8	32
		Degludec	132	60.8 ± 10.0	67(51)	87.1 ± 20.7	31.8 ± 5.7	8.1 ± 0.9	15.1 ± 8.0	
QWINT-1 2025	Argentina, Mexico, and the United States	Efsitora	397	56.4 ± 10.0	203(51.1)	89.3 ± 19.2	32.5 ± 5.8	8.20 ± 0.91	9.2 ± 6.6	52
		Glargine	398	56.2 ± 9.7	195(49.0)	85.5 ± 19.7	31.3 ± 6.1	8.27 ± 1.07	9.6 ± 6.9	
QWINT-3 2025	Argentina, Hungary, Japan, et al	Efsitora	655	62	376(57)	83.6	29.6	7.7	14.5	78
		Degludec	331	62	179(54)	84.3	29.8	7.7	14.4	
QWINT-4 2025	Argentina, Germany, India, et al	Efsitora	365	58.3 ± 10.5	172(47)	87.8 ± 19.9	31.85 ± 5.48	8.36 ± 0.78	16.6 ± 8.8	26
		Glargine	365	59.4 ± 10.5	189(52)	88.4 ± 19.7	31.84 ± 5.48	8.36 ± 0.80	16.9 ± 9.0	
Bajaj 2021	Canada, Czech Republic, Germany, et al	Insulin Icodec	104	62.3 ± 7.7	78(75.0)	NA	29.6 ± 4.2	7.8 ± 0.7	15.2 ± 7.9	16
		Insulin Glargine U100	50	60.5 ± 7.9	33(66.0)	NA	30.3 ± 5.0	7.9 ± 0.7	14.8 ± 8.1	
Lingvay 2021	Croatia, Germany, Hungary, et al	Insulin Icodec	154	60.8 ± 8.4	83(53.9)	89.7 ± 16.6	31.5 ± 4.5	8.1 ± 0.8	9.5 ± 5.6	16
		Insulin Glargine U100	51	60.2 ± 8.1	27(52.9)	86.4 ± 17.1	30.6 ± 4.7	8.2 ± 0.8	11.8 ± 6.8	
NN1436-4383 2020	United States, Canada, Czechia, et al	Insulin Icodec	125	59.7 ± 8.2	70(56.0)	89.7 ± 16.5	31.1 ± 4.9	8.09 ± 0.70	10.5 ± 8.4	26
		Insulin Glargine U100	122	59.4 ± 9.5	69(56.6)	91.3 ± 15.7	31.4 ± 4.4	7.96 ± 0.65	8.8 ± 6.1	
ONWARDS 1 2023	Croatia, India, Israel, et al	Insulin Icodec	492	59.1 ± 10.1	295(60.0)	85.2 ± 17.7	30.0 ± 4.8	8.5 ± 1.0	11.6 ± 6.7	78
		Insulin Glargine U100	492	58.9 ± 9.9	263(53.5)	84.3 ± 17.6	30.1 ± 5.1	8.4 ± 1.0	11.5 ± 6.8	
ONWARDS 2 2023	Bulgaria, Germany, Japan, et al	Insulin Icodec	263	62.3 ± 9.8	162(62)	83.7 ± 18.4	29.5 ± 5.2	8.17 ± 0.77	16.5 ± 8.4	26
		Insulin Degludec	263	62.6 ± 8.4	140(53)	81.5 ± 17.1	29.2 ± 4.9	8.10 ± 0.77	16.9 ± 7.9	
ONWARDS 3 2023	Argentina, Austria, Brazil, et al	Insulin Icodec	294	58 ± 10	185(62.9)	85.8 ± 20.1	29.9 ± 5.2	8.55 ± 1.11	10.5	26
		Insulin Degludec	294	59 ± 10	184(62.6)	83.2 ± 18.2	29.2 ± 5.1	8.48 ± 1.01	10.7	
ONWARDS 4 2023	Belgium, India, Italy, et al	Insulin Icodec	291	59.7 ± 10.1	154(53)	85.5 ± 17.6	30.5 ± 5.0	8.29 ± 0.86	18.0 ± 9.1	26
		Insulin Glargine U100	291	59.9 ± 9.9	150(52)	83.1 ± 17.3	30.0 ± 5.0	8.31 ± 0.90	16.3 ± 7.7	

**Figure 2:**
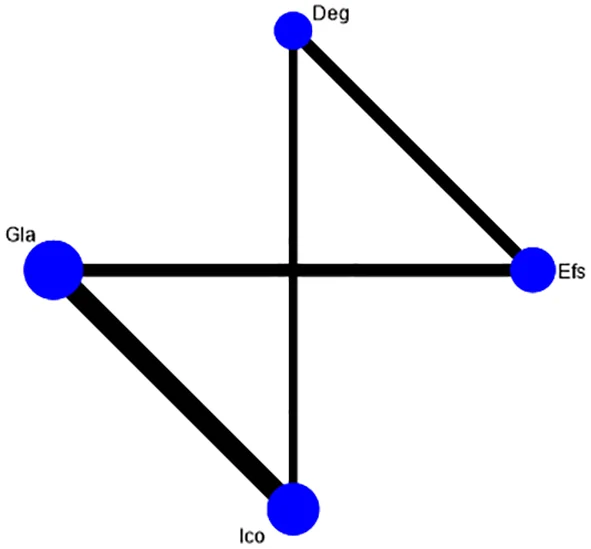
Summary of study retrieval and identification for network meta-analysis. Network plot for all included trials. Deg, insulin degludec; Gla, insulin glargine; Ico, insulin icodec; Efs, insulin efsitora.

The network plot of treatment comparisons is shown in **Figure 2**, where the size of each node is proportional to the total sample size of the corresponding intervention, and the thickness of the edges reflects the number of studies directly comparing the two interventions. As shown in **Figure 2**, the network structure comprises four interventions (insulin icodec, insulin efsitora, insulin glargine, and insulin degludec) connected through multiple direct comparisons. Specifically, direct evidence exists for comparisons between insulin icodec versus insulin glargine, insulin icodec versus insulin degludec, insulin efsitora versus insulin glargine, and insulin efsitora versus insulin degludec. Importantly, no direct head-to-head trial between insulin icodec and insulin efsitora currently exists; therefore, the comparison between these two once-weekly insulins relies entirely on indirect evidence via the common comparators (glargine and degludec). We also performed a consistency assessment using the design-by-treatment interaction model and node-splitting analysis to ensure that indirect estimates were reliable.

### 3.2 Risk of bias and publication bias

**Figure 3:**
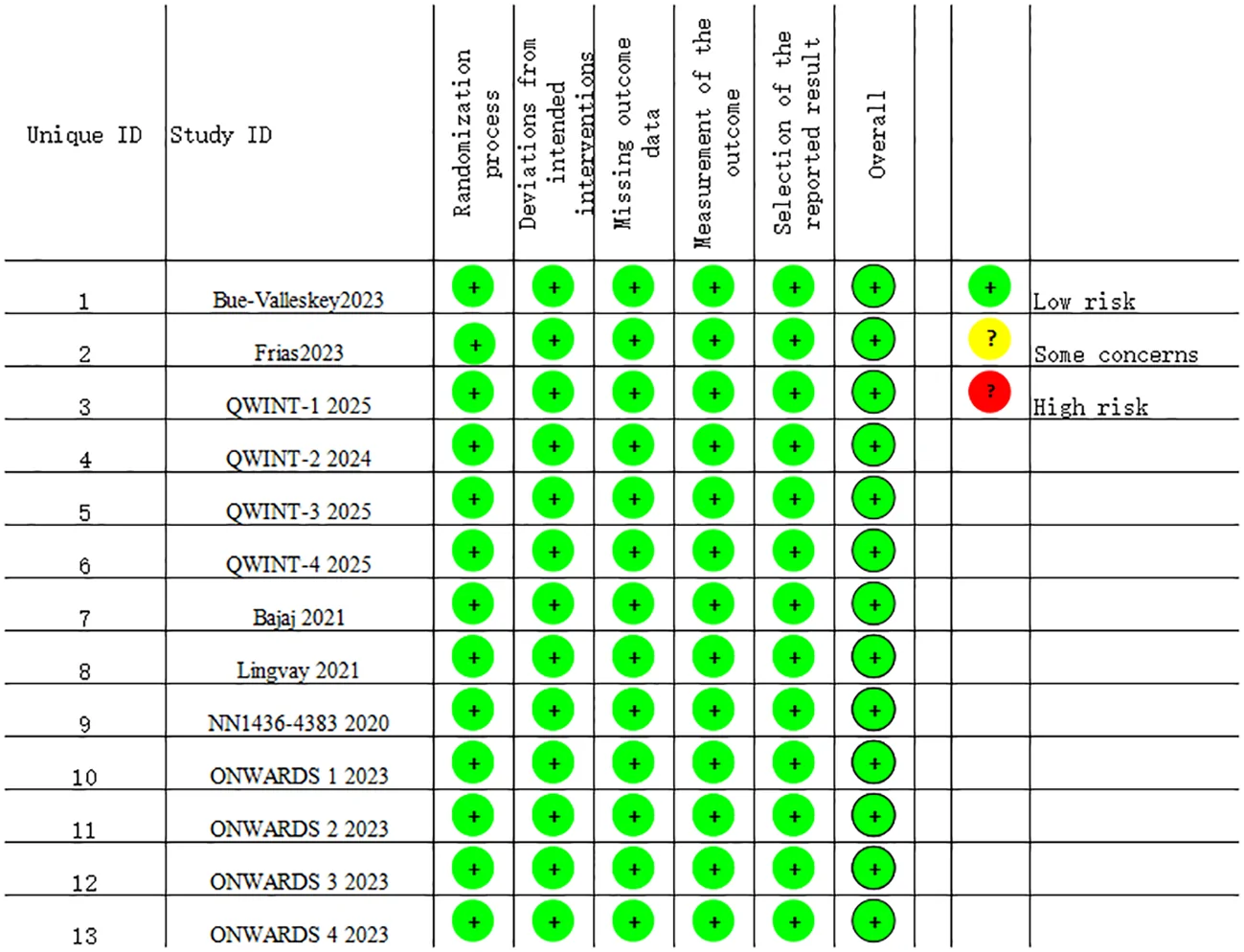
Quality assessment of included studies.

The risk of bias assessment was performed using the Cochrane RoB 2 tool. All included studies were judged as having a low risk of bias (**Figure 3**). Although some studies raised some concerns regarding blinding of participants and personnel due to the open-label design of certain insulin trials, no deviations from the intended intervention occurred, and an appropriate analysis was used to estimate the assignment effect. Therefore, deviations from the intended intervention were rated as low risk.

The evidence classification results, summarized from the GRADE evidence profile assessed by the GRADEpro software, are shown in **Table 2**. The quality of evidence was high for the outcomes of change in HbA1c and change in body weight; moderate for change in FPG, level 1 hypoglycemia, and SAEs; low for level 2 or 3 hypoglycemia and AEs; and very low for the percentage of patients achieving HbA1c < 7%.

**Table 2:** Summary of GRADE evidence profile.

Outcome	Study design	Risk of bias	Inconsistency	Indirectness	Imprecision	Publication bias	Quality of evidence
Change in HbA1c	RCT	No	No	No	No	Undetected	High
Change in body weight	RCT	No	No	No	No	Undetected	High
Percentage of patients achieving HbA1c < 7%	RCT	No	Very serious^1^	No	Serious^2^	Undetected	Very low
Change in FPG	RCT	No	No	No	No	Strongly suspected^5^	Moderate
Level 1 hypoglycemia	RCT	No	No	No	No	Strongly suspected^5^	Moderate
Level 2 or level 3 hypoglycemia	RCT	No	No	No	Serious^3^	Strongly suspected^5^	Low
AEs	RCT	No	No	No	Serious^4^	Strongly suspected^5^	Low
SAEs	RCT	No	No	No	Serious^3^	Undetected	Moderate

1 There is a very serious inconsistency.

2 The RR crosses the line of no effect and spans values that include appreciable benefit.

3 The overall number of events was less than 300.

4 The sample size is less than the optimal information size.

5 Publication bias may exist proved by asymmetrical funnel plot.

### 3.3 Efficacy outcomes

#### 3.3.1 Change in HbA1c

**Figure 4:**
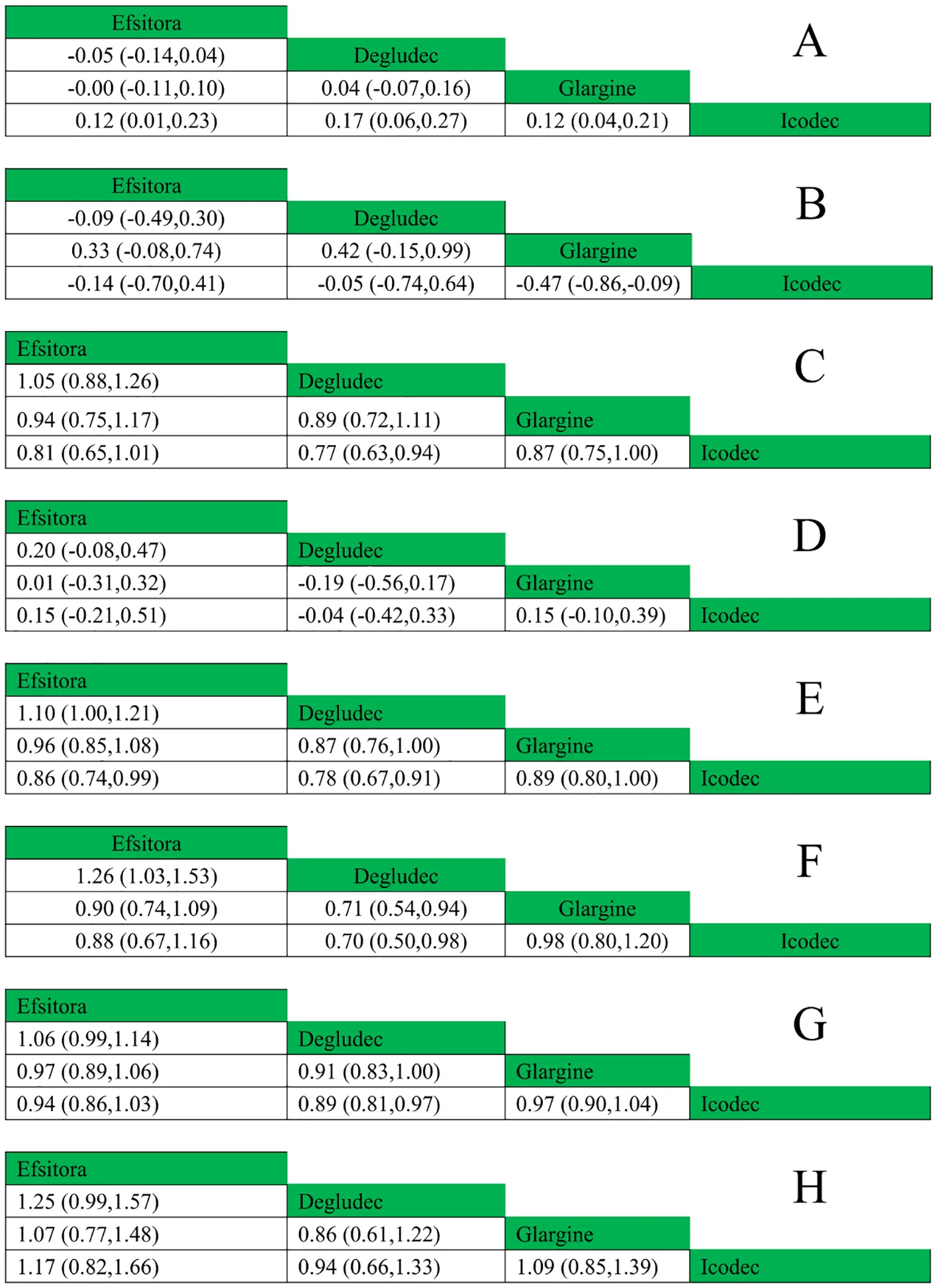
The results of network meta-analysis: **(A)** change in HbA1c, **(B)** change in body weight, **(C)** percentage of patients achieving HbA1c < 7%, **(D)** change in FPG, **(E)** level 1 hypoglycemia, **(F)** level 2 or level 3 hypoglycemia, **(G)** AEs, and **(H)** SAEs.

**Figure 5:**
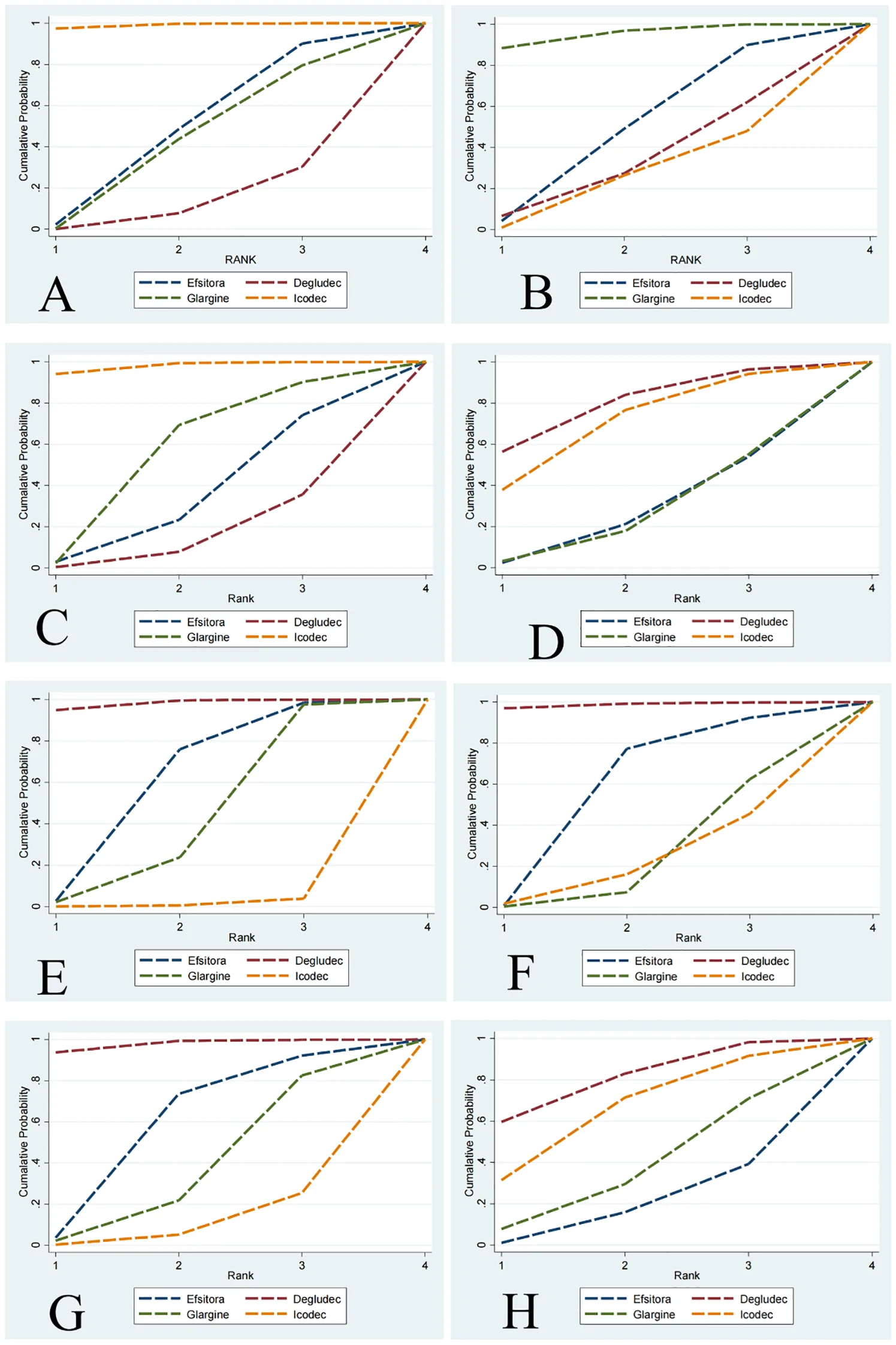
Plot of the SUCRA of safety and efficacy outcomes: **(A)** change in HbA1c, **(B)** change in body weight, **(C)** percentage of patients achieving HbA1c < 7%, **(D)** change in FPG, **(E)** level 1 hypoglycemia, **(F)** level 2 or level 3 hypoglycemia, **(G)** AEs, and **(H)** SAEs.

All 13 RCTs reported data on the change in HbA1c. As shown in **Figure 4A**, insulin icodec demonstrated superior efficacy to insulin efsitora in reducing HbA1c (MD = -0.12%, 95% CI: -0.23% to -0.01%). Insulin icodec resulted in a significantly greater reduction in HbA1c compared with insulin glargine (MD = -0.12%, 95% CI: -0.21% to -0.04%) and insulin degludec (MD = -0.17%, 95% CI: -0.27% to -0.06%). The SUCRA values for HbA1c reduction are presented in **Figure 5A**. The SUCRA results indicated that, among all included interventions, insulin icodec ranked best in terms of change in HbA1c, followed by insulin efsitora, insulin glargine, and insulin degludec.

#### 3.3.2 Change in body weight

A total of 11 studies reported the change in body weight. As shown in **Figure 4B**, the change in body weight was similar between insulin icodec and insulin efsitora (MD = 0.14 kg, 95% CI: −0.41 kg to 0.70 kg). Notably, weight gain was lower with insulin glargine than with insulin icodec (MD = −0.47 kg, 95% CI: −0.86 kg to −0.09 kg). The SUCRA values for body weight change are presented in **Figure 5B**. The SUCRA results indicated that, among all included interventions, insulin glargine ranked best in terms of body weight change, followed by insulin efsitora, insulin degludec, and insulin icodec.

#### 3.3.3 Percentage of patients achieving HbA1c < 7%

Data on the percentage of patients achieving HbA1c <7% were available from 10 trials. As summarized in **Figure 4C**, the likelihood of achieving HbA1c <7% was comparable between insulin icodec and insulin efsitora (RR = 1.23, 95% CI: 0.99-1.54). Notably, a significantly higher proportion of patients in the insulin icodec group achieved this target compared with insulin degludec (RR = 1.30, 95% CI: 1.06-1.58) and insulin glargine (RR = 1.16, 95% CI: 1.00-1.33). The SUCRA values for the percentage of patients achieving HbA1c <7% are presented in **Figure 5C**. The SUCRA results indicated that, among all included interventions, insulin icodec ranked highest in terms of the percentage of patients achieving HbA1c <7%, followed by insulin glargine, insulin efsitora, and insulin degludec.

#### 3.3.4 Change in FPG

A total of 12 studies reported change in FPG. As shown in **Figure 4D**, insulin icodec and insulin efsitora demonstrated comparable effects on FPG reduction (MD = −0.15 mmol/L, 95% CI −0.51 to 0.21 mmol/L). The SUCRA values for change in FPG are presented in **Figure 5D**. The SUCRA results indicated that, among all included interventions, insulin degludec ranked highest in terms of change in FPG, followed by insulin icodec, insulin efsitora, and insulin glargine.

### 3.5 Safety outcomes

#### 3.5.1 Level 1 hypoglycemia

Data on level 1 hypoglycemia were available from 13 studies. As summarized in **Figure 4E**, insulin icodec was associated with a significantly higher incidence of level 1 hypoglycemia compared with insulin efsitora (RR = 1.17, 95% CI: 1.01-1.35), insulin degludec (RR = 1.28, 95% CI: 1.10-1.49), and insulin glargine (RR = 1.12, 95% CI: 1.00-1.26). The SUCRA values for level 1 hypoglycemia are presented in **Figure 5E**. The SUCRA results indicated that, among all included interventions, insulin degludec ranked highest in terms of level 1 hypoglycemia incidence, followed by insulin efsitora, insulin glargine, and insulin icodec.

#### 3.5.2 Level 2 or 3 hypoglycemia

A total of 12 studies reported level 2 or 3 hypoglycemia. As shown in **Figure 4F**, no statistically significant difference was observed between insulin icodec and insulin efsitora for this outcome (RR = 1.14, 95% CI: 0.86-1.50). The incidence of level 2 or 3 hypoglycemia was significantly higher with insulin efsitora (RR = 1.26, 95% CI: 1.03-1.53) and insulin icodec (RR = 1.43, 95% CI: 1.02-2.00) compared with insulin degludec. The SUCRA values for level 2 or 3 hypoglycemia are presented in **Figure 5F**. The SUCRA results indicated that, among all included interventions, insulin degludec ranked highest in terms of level 2 or 3 hypoglycemia incidence, followed by insulin efsitora, insulin glargine, and insulin icodec.

#### 3.5.3 AEs

All 13 studies reported data on overall AEs. As presented in **Figure 4G**, the incidence of any AE was comparable between insulin icodec and insulin efsitora (RR = 1.06, 95% CI 0.97-1.17). The incidence of AEs was significantly higher with insulin icodec (RR = 1.13, 95% CI: 1.03-1.24) compared with insulin degludec. The SUCRA values for AEs are presented in **Figure 5G**. The SUCRA results indicated that, among all included interventions, insulin degludec ranked lowest in terms of AE incidence, followed by insulin efsitora, insulin glargine, and insulin icodec.

#### 3.5.4 SAEs

For SAEs (**Figure 4H**), the incidence was comparable across all treatment groups. Insulin icodec showed no significant difference compared with insulin efsitora (RR = 0.86, 95% CI: 0.60-1.22), and the overall risk of SAEs remained low across all interventions. The SUCRA values for SAEs are presented in **Figure 5H**. These results indicated that, among all included interventions, insulin degludec ranked lowest in terms of SAE incidence, followed by insulin icodec, insulin glargine, and insulin efsitora.

### 3.6 Publication bias

**Figure 6:**
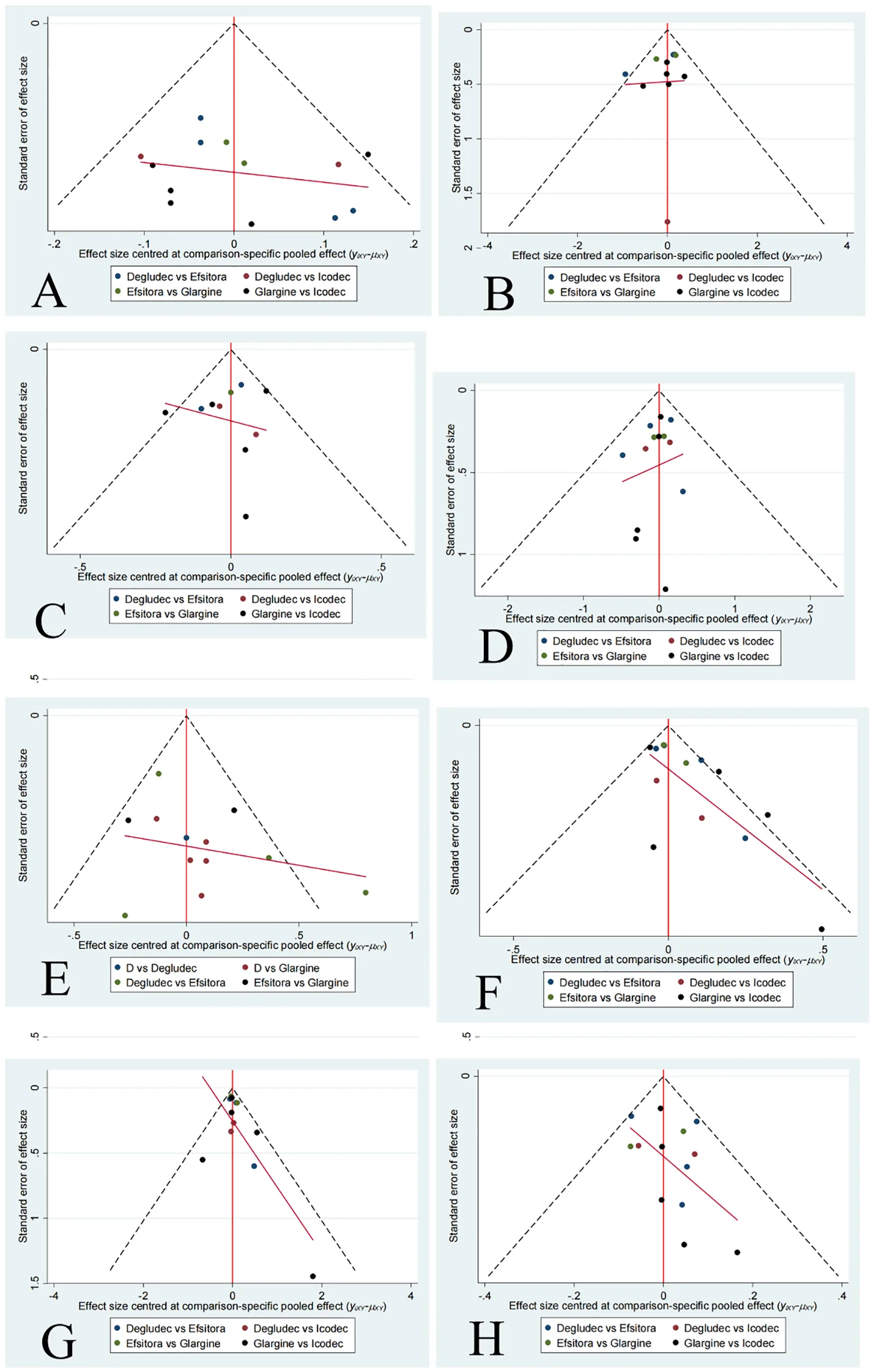
Funnel plot of safety and efficacy outcomes: **(A)** change in HbA1c, **(B)** change in body weight, **(C)** percentage of patients achieving HbA1c < 7%, **(D)** SAEs, **(E)** change in FPG, **(F)** level 1 hypoglycemia, **(G)** level 2 or level 3 hypoglycemia, and **(H)** AEs.

Publication bias was evaluated using comparison-adjusted funnel plots. For change in HbA1c (**Figure 6A**), change in body weight (**Figure 6B**), percentage of patients achieving HbA1c < 7% (**Figure 6C**), and SAEs (**Figure 6D**), the dots were almost symmetrically distributed about the pooled effect size line, indicating the absence of publication bias for these four outcomes. Conversely, for change in FPG (**Figure 6E**), level 1 hypoglycemia (**Figure 6F**), level 2 or 3 hypoglycemia (**Figure 6G**), and AEs (**Figure 6H**), the dots were not symmetrically distributed about the pooled effect size line, suggesting a certain degree of publication bias for these four outcomes.

## 4 Discussion

To our knowledge, this network meta-analysis represents the first comprehensive indirect comparison of the two once-weekly basal insulin analogues, insulin icodec and insulin efsitora, in patients with T2D. In the absence of head-to-head RCTs, our study synthesizes the available evidence from the phase 3 ONWARDS (icodec) and QWINT (efsitora) clinical development programs. The findings suggest that these two novel once-weekly insulins have broadly comparable efficacy and safety profiles, while insulin icodec may provide slightly better glycaemic control than daily insulin.

Although insulin icodec demonstrated a statistically significant reduction in HbA1c compared with insulin efsitora (MD = -0.12%, 95% CI: -0.23% to -0.01%), the magnitude of this difference is modest and likely below the threshold considered clinically meaningful (approximately 0.3%–0.5%). Furthermore, this marginal glycemic advantage should be weighed against the significantly higher incidence of level 1 hypoglycemia observed with insulin icodec. From a clinical standpoint, the two once-weekly insulins offer broadly comparable glycemic efficacy, and treatment selection should be guided by individual patient considerations, including hypoglycemia risk, tolerability, and treatment preference, rather than the small difference in HbA1c reduction.

The development of once-weekly basal insulins directly targets a fundamental challenge in diabetes management: treatment burden and adherence. Daily insulin injections are associated with significant psychological and practical barriers, including fear of injections, the inconvenience of daily dosing schedules, and the perceived complexity of dose titration (28, 29). These factors contribute to clinical inertia and suboptimal persistence with therapy. By reducing the injection frequency from 365 to 52 per year, once-weekly insulins have the potential to alleviate these burdens, improve treatment satisfaction, and enhance long-term adherence.

Insulin icodec and insulin efsitora achieve their prolonged duration of action through distinct molecular strategies. Icodec is an acylated insulin analogue that binds reversibly to albumin, forming an inactive circulating depot from which active insulin is slowly released (6, 30). This mechanism confers a half-life of approximately one week. Efsitora, in contrast, is a fusion protein combining a single-chain insulin variant with a human immunoglobulin G Fc domain, which extends its half-life to approximately 17 days through FcRn-mediated recycling (14, 31). Despite these structural and pharmacokinetic differences, both molecules successfully provide stable, sustained insulin exposure over a one-week dosing interval with relatively flat concentration-time curves. This stability is a key advantage, as it may reduce within-day and between-day glucose variability compared to insulins with sharper peaks.

When compared with traditional daily basal insulins, both once-weekly insulins offer certain advantages. The most obvious benefit is the reduction in injection frequency. However, the prolonged duration of action also presents unique clinical considerations. Once a weekly dose is administered, its effects persist for several days, meaning that dose adjustments take longer to manifest their full impact on glucose levels. This property necessitates careful attention to initial dose selection and titration, particularly when initiating therapy or adjusting doses in response to hypoglycemia. In contrast, daily insulins allow for more frequent, granular dose adjustments, which may be advantageous in patients with variable eating patterns, fluctuating insulin requirements, or heightened susceptibility to hypoglycemia.

The higher incidence of level 1 hypoglycemia with insulin icodec compared with insulin efsitora is likely multifactorial. Pharmacokinetically, icodec’s approximately one−week half−life and albumin−binding profile may yield slightly more variable glucose exposure than efsitora’s longer 17−day half−life and flatter concentration−time curve via FcRn recycling. Differences in titration algorithms between the ONWARDS and QWINT programs-potentially more aggressive with icodec to achieve greater HbA1c reduction-may have increased hypoglycemic events. Additionally, the open−label design of most trials may have introduced detection bias, as patients aware of their treatment could report minor hypoglycemia more frequently. Variations in concomitant sulfonylurea use and other baseline factors may also have contributed, though these were not fully detailed. Thus, the observed difference reflects a combination of pharmacokinetic properties, titration strategies, study design limitations, and patient−related variables, underscoring the need for individualized patient selection and careful dose management when using once−weekly insulins.

Recent treatment recommendations have emphasized that body weight control should be a priority in the management of T2D (32, 33). In contrast, once-weekly insulins are generally weight-neutral or associated with modest weight gain, and they do not carry the same gastrointestinal side effect burden. Therefore, the choice between a once-weekly insulin and a once-weekly GLP-1/GIP receptor agonist should be guided by patient-specific factors, including the degree of hyperglycemia, the need for weight loss, tolerability, and patient preference (34, 35). For patients who require substantial glycemic improvement, have contraindications to or intolerance of GLP-1 receptor agonists, or have advanced T2D with significant insulin deficiency, once-weekly basal insulins represent a valuable and convenient option.

To support the validity of our indirect comparisons, we carefully evaluated the comparability of patient populations across the ONWARDS (icodec) and QWINT (efsitora) programs. As summarized in **Table 1**, the baseline characteristics were broadly similar across trials. Mean age ranged from 56.2 to 62.6 years, mean baseline HbA1c ranged from 7.7% to 8.55%, mean BMI ranged from 29.2 to 32.5 kg/m², and mean duration of diabetes ranged from 8.8 to 18.0 years. The proportion of male participants, background glucose-lowering therapies, and treatment durations were also generally consistent across studies. These similarities support the transitivity assumption and suggest that the indirect comparison between insulin icodec and insulin efsitora is unlikely to be substantially confounded by differences in patient characteristics. Nevertheless, we acknowledge that unmeasured or unreported effect modifiers, such as prior insulin use patterns and local clinical practices, may introduce residual heterogeneity, and our findings should be interpreted with appropriate caution.

Longer treatment durations, such as those in QWINT-3 (78 weeks) and ONWARDS 1 (78 weeks), may allow for more complete dose titration, greater cumulative glycemic exposure, and more stable long-term glycemic control, potentially leading to larger HbA1c reductions and a higher proportion of patients achieving HbA1c <7%. Conversely, shorter trials (e.g., 16 weeks in Bajaj 2021 and Lingvay 2021) may capture early-phase effects and titration-related hypoglycemia more prominently, as doses are being adjusted more aggressively during the initial weeks. For safety outcomes, longer follow-up provides more opportunity to observe AEs and hypoglycemic episodes, which could partly explain the higher event rates in some extended-duration studies. Additionally, the open-label design of most trials means that the duration of exposure may influence patient and investigator behavior, particularly regarding hypoglycemia reporting and dose adjustment decisions.

Potential publication bias was observed for several outcomes, namely change in FPG, level 1 hypoglycemia, level 2 or 3 hypoglycemia, and AEs. Asymmetry in the comparison-adjusted funnel plots for these outcomes suggests that smaller studies with non-significant or negative results may be underrepresented in the published literature, which could lead to overestimation of treatment effects or underestimation of safety risks. For instance, if trials reporting higher hypoglycemia rates with insulin icodec were less likely to be published, our observed RRs for level 1 hypoglycemia might be conservative; conversely, if smaller trials with favorable results were overrepresented, the apparent superiority of icodec in HbA1c reduction could be exaggerated. It is also worth noting that publication bias is more common in industry-sponsored trials, which constitute the majority of the included studies, and may reflect selective reporting of positive outcomes.

As shown in **Table 1**, the included trials enrolled both subgroups-insulin-naive patients (e.g., ONWARDS 1, ONWARDS 3, QWINT-1, QWINT-2) and insulin-experienced patients (e.g., ONWARDS 2, ONWARDS 4, QWINT-3, QWINT-4)-and our pooled results represent average treatment effects across this diverse population. However, we acknowledge that the findings may not be equally applicable to both groups, as insulin-naive patients may derive greater adherence benefits from once-weekly dosing, while insulin-experienced patients with longer diabetes duration and prior hypoglycemia history may have different baseline risks and responses. The generalizability of our findings to specific patient subgroups should be interpreted with caution, and that future head-to-head trials with predefined subgroup analyses are warranted to confirm whether the comparative profiles differ according to prior insulin exposure.

The findings of this network meta-analysis have several important implications for clinical practice. First, for patients with T2D who require initiation or intensification of basal insulin therapy and who strongly prefer to minimize injection frequency, both insulin icodec and insulin efsitora are effective and safe options. Their comparable profiles suggest that treatment selection may be guided by factors such as local availability, formulary coverage, cost, patient preference, and clinician familiarity with the specific titration algorithm. Second, the choice between once-weekly and daily basal insulins should be highly individualized. Patients with a history of recurrent severe hypoglycemia, impaired awareness of hypoglycemia, advanced age, frailty, significant renal impairment, or erratic eating patterns may be better served by daily insulins that allow for more frequent and granular dose adjustments. Conversely, patients who are stable, adherent, and motivated to simplify their regimen may derive substantial benefit from once-weekly dosing. Third, proper patient education is paramount. Patients initiating once-weekly insulin must understand that the long half-life means that dose adjustments take time to manifest their full effect and that persistent hyperglycemia should not be managed by unscheduled additional doses. Conversely, patients must be educated about the signs, symptoms, and management of hypoglycemia and advised to monitor their blood glucose levels regularly, particularly during the titration phase. Fourth, concomitant medications should be reviewed and optimized when initiating once-weekly insulin. The use of sulfonylureas may potentiate the risk of hypoglycemia and can often be discontinued or reduced. Conversely, the concomitant use of SGLT2 inhibitors or GLP-1 receptor agonists may complement once-weekly insulin by improving glycemic control and promoting weight loss, although clinicians should be aware of potential additive risks.

This study has several strengths. It is the first network meta-analysis to directly compare insulin icodec and insulin efsitora, addressing an important evidence gap. The inclusion of a large number of RCTs with a substantial total participant population provides a robust evidence base. The comprehensive assessment of multiple efficacy and safety outcomes offers a holistic view of the benefit-risk profile of these agents. The use of a rigorous frequentist network meta-analysis framework, with careful attention to assumptions of transitivity, consistency, and heterogeneity, enhances the reliability of our findings.

However, several limitations should be acknowledged. First, the absence of direct head-to-head RCTs between icodec and efsitora means that our comparisons are indirect, relying on common comparators (glargine, or degludec). While network meta-analysis is a well-established methodology, the results should be interpreted with appropriate caution. Second, clinical and methodological heterogeneity (e.g., titration heterogeneity) existed across the included trials. Third, the open-label design of most included studies introduces the potential for performance and detection bias, particularly for patient-reported outcomes such as level 1 hypoglycemia and level 2 or 3 hypoglycemia. Fourth, the generalizability of our findings to specific subpopulations (e.g., elderly patients, those with severe renal impairment, or non-White ethnic groups) may be limited due to underrepresentation of these groups in some trials. Finally, we did not formally assess patient-reported outcomes such as quality of life, treatment burden, or injection-related anxiety, which are important considerations given the primary rationale for once-weekly insulin therapy. Future research should prioritize these patient-centered endpoints.

## 5 Conclusions

In conclusion, this network meta-analysis reveals that insulin icodec and insulin efsitora have distinct benefit-risk profiles in patients with T2D. Insulin icodec provided a modest but statistically significant reduction in HbA1c compared with insulin efsitora; however, this glycemic advantage was accompanied by a significantly higher incidence of level 1 hypoglycemia. For other outcomes-including change in body weight, percentage of patients achieving HbA1c <7%, change in FPG, incidence of level 2 or 3 hypoglycemia, AEs, and SAEs-the two agents showed no statistically significant differences. Compared with traditional daily basal insulins, insulin icodec provided superior HbA1c reduction and a higher likelihood of reaching HbA1c < 7%, whereas insulin degludec had the most favourable hypoglycaemia profile. Direct head to head RCTs are warranted to confirm these findings. Future studies evaluating patient-reported outcomes, treatment satisfaction, and adherence may provide additional clinically relevant insights.

## Data Availability

The original contributions presented in the study are included in the article/supplementary material. Further inquiries can be directed to the corresponding author/s.

## References

[1] Magliano DJ Boyko E Balkau B International Diabetes Federation (Brussels) . IDF Diabetes Atlas 2021—10th Edition (2021). Available online at: https://international-diabetes-federation.s3.eu-west-1.amazonaws.com/media/uploads/sites/3/2025/02/IDF_Atlas_10th_Edition_2021.pdf (Accessed June 5, 2026). 35914061

[2] Okemah J Peng J Quiñones M . Addressing clinical inertia in type 2 diabetes mellitus: a review. Adv Ther. (2018) 35:1735–45. doi: 10.1007/s12325-018-0819-5 30374807 PMC6223992

[3] Ross SA Tildesley HD Ashkenas J . Barriers to effective insulin treatment: the persistence of poor glycemic control in type 2 diabetes. Curr Med Res Opin. (2011) 27:13–20. doi: 10.1185/03007995.2011.621416 21942467

[4] Peyrot M Rubin RR Kruger DF Travis LB . Correlates of insulin injection omission. Diabetes Care. (2010) 33:240–5. doi: 10.2337/dc09-1348 20103556 PMC2809256

[5] Peyrot M Barnett AH Meneghini LF Schumm-Draeger PM . Insulin adherence behaviours and barriers in the multinational Global Attitudes of Patients and Physicians in Insulin Therapy study. Diabetes Med. (2012) 29:682–9. doi: 10.1111/j.1464-5491.2012.03605.x 22313123 PMC3433794

[6] Nishimura E Pridal L Glendorf T Hansen BF Hubálek F Kjeldsen T . Molecular and pharmacological characterization of insulin icodec: a new basal insulin analog designed for once-weekly dosing. BMJ Open Diabetes Res Care. (2021) 9:e002301. doi: 10.1136/bmjdrc-2021-002301 34413118 PMC8378355

[7] Kazda CM Bue-Valleskey JM Chien J Zhang Q Chigutsa E Landschulz W . Novel once-weekly basal insulin Fc achieved similar glycemic control with a safety profile comparable to insulin degludec in patients with type 1 diabetes. Diabetes Care. (2023) 46:1052–9. doi: 10.2337/dc22-2395 36920867 PMC10154655

[8] Rosenstock J Bain SC Gowda A Jódar E Liang B Lingvay I . Weekly icodec versus daily glargine U100 in type 2 diabetes without previous insulin. N Engl J Med. (2023) 389:297–308. doi: 10.1056/nejmoa2303208 37356066

[9] Philis-Tsimikas A Asong M Franek E Jia T Rosenstock J Stachlewska K . Switching to once-weekly insulin icodec versus once-daily insulin degludec in individuals with basal insulin-treated type 2 diabetes (ONWARDS 2): a phase 3a, randomised, open label, multicentre, treat-to-target trial. Lancet Diabetes Endocrinol. (2023) 11:414–25. doi: 10.1016/s2213-8587(23)00093-1 37148899

[10] Lingvay I Asong M Desouza C Gourdy P Kar S Vianna A . Once-weekly insulin icodec vs once-daily insulin degludec in adults with insulin-naive type 2 diabetes: the ONWARDS 3 randomized clinical trial. JAMA. (2023) 330:228–37. doi: 10.1001/jama.2023.11313 37354562 PMC10354685

[11] Mathieu C Ásbjörnsdóttir B Bajaj HS Lane W Matos ALSA Murthy S . Switching to once-weekly insulin icodec versus once-daily insulin glargine U100 in individuals with basal-bolus insulin-treated type 2 diabetes (ONWARDS 4): a phase 3a, randomised, open-label, multicentre, treat-to-target, non-inferiority trial. Lancet. (2023) 401:1929–40. doi: 10.1016/s0140-6736(23)00520-2 37156252

[12] Bajaj HS Aberle J Davies M Donatsky AM Frederiksen M Yavuz DG . Once-weekly insulin icodec with dosing guide app versus once-daily basal insulin analogues in insulin-naive type 2 diabetes (ONWARDS 5): a randomized trial. Ann Intern Med. (2023) 176:1476–85. doi: 10.7326/m23-1288 37748181

[13] Russell-Jones D Babazono T Cailleteau R Engberg S Irace C Kjaersgaard MIS . Once-weekly insulin icodec versus once-daily insulin degludec as part of a basal-bolus regimen in individuals with type 1 diabetes (ONWARDS 6): a phase 3a, randomised, open-label, treat-to-target trial. Lancet. (2023) 402:1636–47. doi: 10.1016/s0140-6736(23)02179-7 37863084

[14] Heise T Chien J Beals JM Benson C Klein O Moyers JS . Pharmacokinetic and pharmacodynamic properties of the novel basal insulin Fc (insulin efsitora alfa), an insulin fusion protein in development for once-weekly dosing for the treatment of patients with diabetes. Diabetes Obes Metab. (2023) 25:1080–90. doi: 10.1111/dom.14956 36541037

[15] Rosenstock J Bailey T Connery L Miller E Desouza C Wang Q . Weekly fixed-dose insulin efsitora in type 2 diabetes without previous insulin therapy. N Engl J Med. (2025) 393:325–35. doi: 10.1056/nejmoa2502796 40548694

[16] Wysham C Bajaj HS Del Prato S Franco DR Kiyosue A Dahl D . Insulin efsitora versus degludec in type 2 diabetes without previous insulin treatment. N Engl J Med. (2024) 391:2201–11. doi: 10.1056/nejmoa2403953 39254740

[17] Philis-Tsimikas A Bergenstal RM Bailey TS Jinnouchi H Thrasher JR Ilag L . Once-weekly insulin efsitora alfa versus once-daily insulin degludec in adults with type 2 diabetes currently treated with basal insulin (QWINT-3): a phase 3, randomised, non-inferiority trial. Lancet. (2025) 405:2279–89. doi: 10.1016/s0140-6736(25)01044-x 40562047

[18] Blevins T Dahl D Pérez Manghi FC Murthy S Ortiz Carrasquillo R Li X . Once-weekly insulin efsitora alfa versus once-daily insulin glargine U100 in adults with type 2 diabetes treated with basal and prandial insulin (QWINT-4): a phase 3, randomised, non-inferiority trial. Lancet. (2025) 405:2290–301. doi: 10.1016/s0140-6736(25)01069-4 40562048

[19] Bergenstal RM Weinstock RS Mathieu C Onishi Y Vijayanagaram V Katz ML . Once-weekly insulin efsitora alfa versus once-daily insulin degludec in adults with type 1 diabetes (QWINT-5): a phase 3 randomised non-inferiority trial. Lancet. (2024) 404:1132–42. doi: 10.1016/s0140-6736(24)01804-x 39270686

[20] Hammad N Abdelaziz A Abouzkaly F Saad OA Elazab A Atta K . Comparative effectiveness of once-weekly insulin efsitora alfa in patients with type 2 diabetes: a systematic review and meta-analysis of randomized controlled trials. Diabetes Obesity CardioMetabolic Care. (2026) 1:267–77. doi: 10.2337/doc25-0066

[21] Soetedjo NNM Permana H Hariyanto TI Tendean M Kusumawati M Ritonga E . Once-weekly insulin icodec as novel treatment for type 2 diabetes mellitus: a systematic review and meta-analysis of randomized clinical trials. Diabetes Res Clin Pract. (2023) 205:110984. doi: 10.1016/j.diabres.2023.110984 39445433

[22] Hutton B Salanti G Caldwell DM Chaimani A Schmid CH Cameron C . The PRISMA extension statement for reporting of systematic reviews incorporating network meta-analyses of health care interventions: checklist and explanations. Ann Intern Med. (2015) 162:777–84. doi: 10.7326/m14-2385 26030634

[23] Bajaj HS Bergenstal RM Christoffersen A Davies MJ Gowda A Isendahl J . Switching to once-weekly insulin icodec versus once-daily insulin glargine U100 in type 2 diabetes inadequately controlled on daily basal insulin: a phase 2 randomized controlled trial. Diabetes Care. (2021) 44:1586–94. doi: 10.2337/dc20-2877 33875485 PMC8323191

[24] Bue-Valleskey JM Kazda CM Ma C Chien J Zhang Q Chigutsa E . Once-weekly basal insulin Fc demonstrated similar glycemic control to once-daily insulin degludec in insulin-naive patients with type 2 diabetes: a phase 2 randomized control trial. Diabetes Care. (2023) 46:1060–7. doi: 10.2337/dc22-2396 36944059 PMC10154646

[25] Frias J Chien J Zhang Q Chigutsa E Landschulz W Syring K . Safety and efficacy of once-weekly basal insulin Fc in people with type 2 diabetes previously treated with basal insulin: a multicentre, open-label, randomised, phase 2 study. Lancet Diabetes Endocrinol. (2023) 11:158–68. doi: 10.1016/s2213-8587(22)00388-6 36758572

[26] Lingvay I Buse JB Franek E Hansen MV Koefoed MM Mathieu C . A randomized, open-label comparison of once-weekly insulin icodec titration strategies versus once-daily insulin glargine U100. Diabetes Care. (2021) 44:1595–603. doi: 10.2337/figshare.14054921 33875484 PMC8323172

[27] Rosenstock J Bajaj HS Janež A Silver R Begtrup K Hansen MV . Once-weekly insulin for type 2 diabetes without previous insulin treatment. N Engl J Med. (2020) 383:2107–16. doi: 10.1056/nejmoa2022474 32960514

[28] Gelsey FT Schapiro D Kosa K Vass C Perez-Nieves M Pierce A . Perspectives and preferences of people with type 2 diabetes for the attributes of weekly insulin. Diabetes Ther. (2024) 15:2367–79. doi: 10.1007/s13300-024-01652-0 39347898 PMC11467135

[29] Polonsky WH Fisher L Hessler D Bruhn D Best JH . Patient perspectives on once-weekly medications for diabetes. Diabetes Obes Metab. (2011) 13:144–9. doi: 10.1111/j.1463-1326.2010.01327.x 21199266

[30] Kjeldsen TB Hubálek F Hjørringgaard CU Tagmose TM Nishimura E Stidsen CE . Molecular engineering of insulin icodec, the first acylated insulin analog for once-weekly administration in humans. J Med Chem. (2021) 64:8942–50. doi: 10.1021/acs.jmedchem.1c00257 33944562

[31] Moyers JS Hansen RJ Day JW Dickinson CD Zhang C Ruan X . Preclinical characterization of LY3209590, a novel weekly basal insulin Fc-fusion protein. J Pharmacol Exp Ther. (2022) 382:346–55. doi: 10.1124/jpet.122.001105 35840338

[32] Davies MJ Aroda VR Collins BS Gabbay RA Green J Maruthur NM . Management of hyperglycaemia in type 2 diabetes, 2022. A consensus report by the American Diabetes Association (ADA) and the European Association for the Study of Diabetes (EASD). Diabetologia. (2022) 65:1925–66. doi: 10.2337/figshare.20800537.v1 PMC9510507 36151309

[33] American Diabetes Association Professional Practice Committee . 8. Obesity and weight management for the prevention and treatment of type 2 diabetes: standards of care in diabetes-2024. Diabetes Care. (2024) 47:S145–57. doi: 10.2337/dc24-S008 PMC10725806 38078578

[34] Gorgojo-Martínez JJ Mezquita-Raya P Carretero-Gómez J Castro A Cebrián-Cuenca A de Torres-Sánchez A . Clinical recommendations to manage gastrointestinal adverse events in patients treated with GLP-1 receptor agonists: a multidisciplinary expert consensus. J Clin Med. (2022) 12:145. doi: 10.3390/jcm12010145 36614945 PMC9821052

[35] Ayesh H Suhail S Ayesh S Niswender K . Comparative efficacy and safety of weekly tirzepatide versus weekly insulin in type 2 diabetes: a network meta-analysis of randomized clinical trials. Diabetes Obes Metab. (2024) 26:3801–9. doi: 10.1111/dom.15725 38923379

